# Emergency physician personnel crisis: a survey on attitudes of new generations in Slovenia

**DOI:** 10.1186/s12873-024-00940-z

**Published:** 2024-02-14

**Authors:** Luka Petravić, Boštjan Bajec, Evgenija Burger, Eva Tiefengraber, Ana Slavec, Matej Strnad

**Affiliations:** 1 Center for Emergency Medicine, University Medical Center Maribor, Ljubljanska ulica 5, 2000 Maribor, Slovenia; 2 Department of Psychology, Faculty of Arts, University of Ljubljana, Aškerčeva 2, 1000 Ljubljana, Slovenia; 3 Faculty of Mathematics and Physics, University of Ljubljana, Jadranska ulica 19, 1000 Ljubljana, Slovenia; 4 Faculty of Medicine, University of Maribor, Taborska ulica 8, 2000 Maribor, Slovenia; 5 InnoRenew CoE, Livade 6a, 6310 Izola, Slovenia; 6 Department of Applied Natural Sciences, University of Primorska, Glagoljaška 8, 6000 Koper, Slovenia; 7 Community healthcare center dr. Adolf Drolca, Prehospital unit, Ulica talcev 9, 2000 Maribor, Slovenia

**Keywords:** Internship and residency, Slovenia, Students, Workforce, Surveys and questionnaires, Salaries and Fringe benefits, Policy

## Abstract

**Background:**

Emergency departments globally are overburdened, and emergency medicine residency is losing popularity among students and physicians. This raises concerns about the collapse of a life-saving system. Our goal was to identify the key workforce reasoning and question medical staff employment behavior.

**Methods:**

This was a prospective cross-sectional study. In December 2022, medical students and pre-residency doctors in Slovenia were invited to complete a web-based questionnaire. The data were analyzed using T-test, chi-square test, Mann‒Whitney-Wilcoxon tests, and principal component analysis. Open-ended questions were hand-categorized.

**Results:**

There were 686 participatns who clicked on the first page and 436 of those finished the survey. 4% of participants gave a clear positive response, while 11% responded positively regarding their decision to pursue emergency medicine residency. The popularity of emergency medicine decreases significantly among recent medical school graduates upon their initial employment. People who choose emergency medicine are less concerned about its complexity and pressure compared to others. Most respondents preferred 12-hour shift lengths. The preferred base salary range for residents was I$ 3623–4529, and for specialists, it was I$ 5435–6341. The sample’s primary personal priorities are achieving a satisfactory work-life balance, earning respect from colleagues, and engaging in academic activities. Factors that attract individuals to choose emergency medicine include high hourly wages, establishment of standards and norms, and reduced working hours.

**Conclusions:**

Our findings indicate that enhancing compensation, establishing achievable standards and norms, facilitating a beneficial work-life equilibrium, providing assistance with initial property acquisition, stimulating participation in deficit residency programs, fostering collegiality among peers, restricting the duration of shifts, and enabling pension accrual may be imperative in attracting more individuals to pursue emergency medicine residency.

**Supplementary Information:**

The online version contains supplementary material available at 10.1186/s12873-024-00940-z.

## Background

Social cognitive career theory [1] explains the interplay between personal factors, environmental factors, and behaviors of individuals in career development and career choice. It is based on Bandura’s social cognitive theory [2], which builds on the triadic reciprocity of the individual, the individual’s behavior, and the environment [3] to explain human behavior and learning. The theory states that beliefs about behavior and goals that people set about their careers guide their behavior, which affects their beliefs and goals. Various types of outcome expectations (in addition to self-efficacy beliefs and personal factors) help foster interests that can guide choices in a career [1]. These outcome expectations can be affected by labor supply and demand [4, 5], underemployment, and wages [5], among other factors.

Previous studies [6–8] have dealt with factors influencing medical students’ choice of specialization; however, research in former communist countries and Central European countries is rather scarce [7, 9, 10], and none of them dealt with the emergency medicine field, which is especially understaffed in Slovenia [11] as well as elsewhere [12].

Emergency medicine (EM) is one of the main pillars of primary health care and is responsible for handling a high number of patients daily [13]. It encompasses a wide range of services, from minor injury management to critical care, spanning primary, secondary, and tertiary care [12]. Consequently, this field requires a substantial number of EM physicians, among other staff.

EM residency in Slovenia was established in 2008, with a residency length of 5 years [13]. Sixteen European countries have recognized EM as a specialty requiring at least 5 years of training time, whereas 17 offer a 4-year curriculum or a 2-3-year subspecialty program [14]. In 2022, the Medical Chamber of Slovenia’s registry listed 185 active EM specialists, equating to 0.0877 professionals per 1000 inhabitants, placing Slovenia near the bottom among European countries in this regard [11]. In 2022, 11 candidates applied for EM residency in Slovenia, whereas in 2023, only 3 out of 17 positions have been filled [11]. While EM is growing and flourishing globally, the last decade has seen the accumulation of workforce problems [12]. Young doctors in Slovenia show less interest in EM residency due to disorganized working conditions, an unclear future for EM physicians, and excessive workloads in emergency departments (EDs) [13]. EDs are very stressful and complex environments. Since 1997, ED visits have increased by 60%, and during the COVID-19 epidemic, these numbers have risen even further [15].

### Slovenia

#### Country and health system

**Fig. 1:**
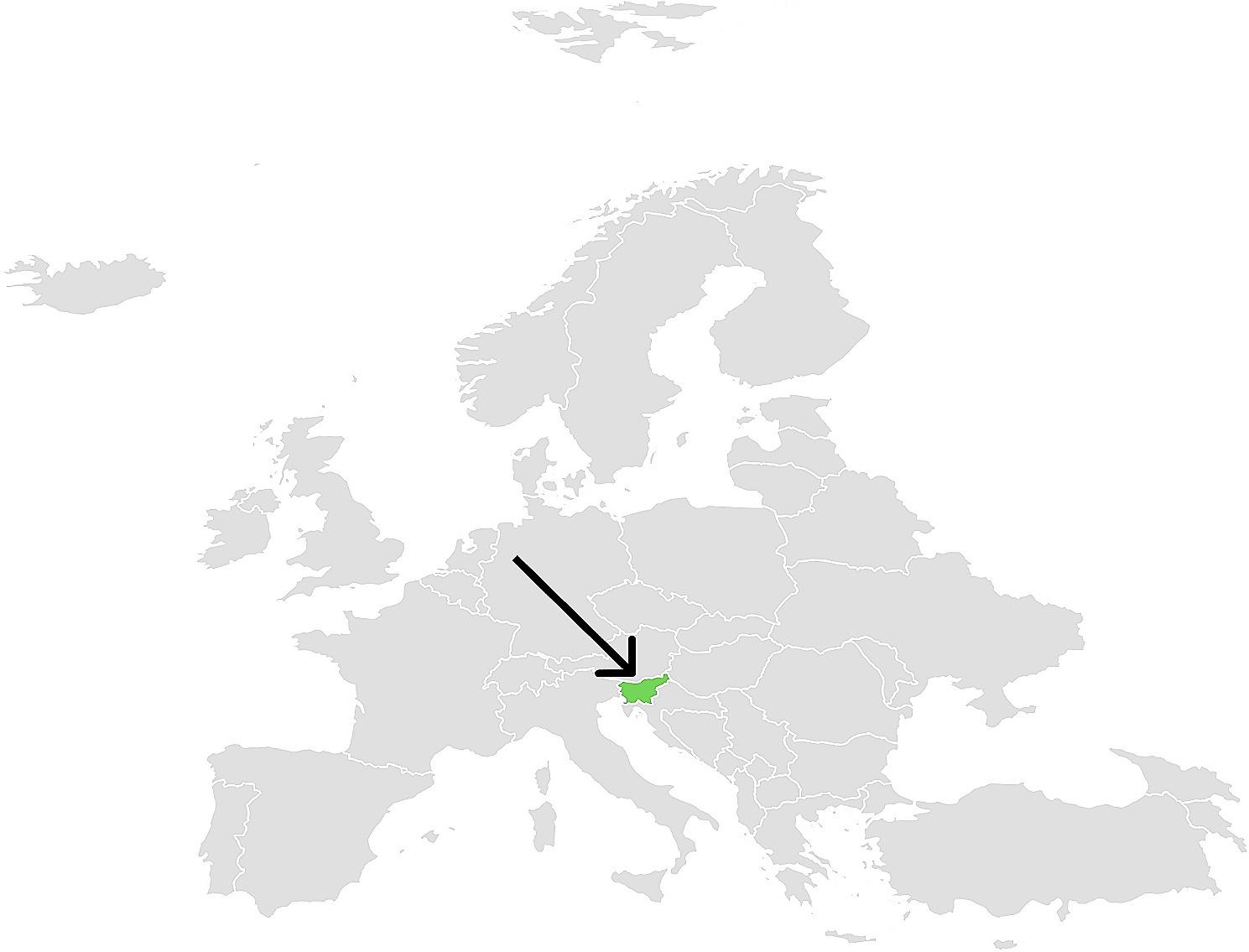
Map of Europe with Slovenia marked green and with a black arrow

Slovenia is a country with over 2 million inhabitants and is the most economically developed of the post-Communist countries to join the European Union, entring the Euro zone in 2007 [16]. Geographically, Slovenia is located between the Alps, the Pannonian Plain, the Mediterranean Sea and the Balkans (Fig. 1) [16]. It borders Austria, Hungary, Italy and Croatia. The majority of its population, over 80%, are Slovenes, a Slavic ethnic group [16]. The Slovenian political system is a parliamentary democracy, with powers divided among judiciary, executive and legislative authorities [16]. In 2022, Slovenia’s GDP per capita was US$ 48,362, with health expenditures of US$ 4,114 per capita and an annual average wage of US$ 47,203.6 [17].

#### Helthcare in Slovenia

Slovenia operates a Bismarck-type social health insurance system with a single public insurer, the Health Insurance Institute of Slovenia (HIIS) [16]. The Ministry of Health is the owner, key investor and manager of all public hospitals [16]. Primary healthcare, on the other hand, is overseen by local municipalities, totaling 212 [16]. Since 1992, the healthcare system has undergone a transformation into a mixed system where private sources of funding have become significant [16].

#### Medical schools in Slovenia

Higher education in Slovenia is funded by the taxpayers, with no copayment needed from the students. There are currently two medical schools in Slovenia, located in the country’s two largest cities. The Faculty of Medicine Ljubljana, established in 1919, is the larger of the two [18]. Over the years, it has educated over 9,000 physicians [18]. In 2022, the faculty admitted approximately 270 first-year students, a significant increase compared to 150 students in their 6th year. The Faculty of Medicine Maribor, established in 2003, was recognized as a necessity as early as 1960 by the Faculty of Medicine Ljubljana [19]. The cohorts in Maribor are smaller, with the 2022 first-year class comprising approximately 120 students and the 6th -year class having 76 students.

The curriculum is similar between the two faculties, with the main difference being the commencement of clinical work. In Maribor, clinical work begins in the 3rd year, compared to the 4th year in Ljubljana. The program takes 6 years to complete, and the diploma is recognized throughout Europe.

#### Career pathway of Slovenian doctors

**Fig. 2:**
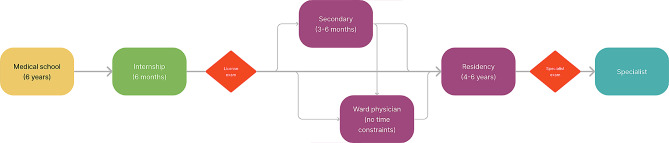
Path from medical school to a specialist in Slovenia

All doctors wishing to work in health care in Slovenia must become members of the Medical Chamber to do so [16]. There is only one medical chamber, and it requires a monthly membership fee. After graduating from medical school, junior doctors are needed to complete a six-month internship focusing on intensive care and emergency medicine. This internship has been compulsory since 2007 [16]. Following the internship, they must pass a licensing exam to gain the right to enter structured residency training (Fig. 2). After obtaining their license, they have several options: they can undertake a secondary rotation, which allows them to experience different residencies before committing to one; they can work as ward physicians, managing patient care on wards before applying for residency; or they can apply directly for a specific residency. To match a residency, a candidate applies to 3 different residency openings. The first choice is their preferred match, while the second and third choices serve as alternatives in case the first option is unavailable.

#### Doctor salaries

Most doctors are employed in the public health system, making them part of the public wage system organized into classes. There are 66 wage classes in total. The minimal wage is set higher than the 1st wage class, resulting in the minimum wage being paid out with the 25th wage class. In 2022, the average gross monthly wage was I$ 3613, while the minimal wage was I$ 1922. An intern doctor earns I$ 2670, a first-year resident earns I$ 3513, and a specialist’s starting salary is I$ 5000. The highest net base salary that can be achieved is I$ 7402. These figures do not include compensation for overtime, special working conditions, or work during weekends and holidays. The minimum workweek is set at 40 h; officially, the maximum workweek is set at 48 h, but this has yet to be achieved [20].

#### Aim of the study

With EDs being understaffed, it is crucial to identify the attitudes of medical students and young doctors before they apply for residency. This approach can help offer favorable working conditions to alleviate a chronic doctor shortage and prevent subsequent ED collapse. The survey aimed to gather information from presidency individuals regarding the opportunities for residency in EM to inquire about their employment behavior and to identify the reasoning behind their choices. It seeks to understand their attitudes and what could influence them to choose EM residency [21].

## Methods

### Study description

The study methodology is described according to the Checklist for Reporting Results of Internet E-Surveys (CHERRIES) checklist [22]. The survey was designed to gather the attitudes and opinions of Slovenian medical students and junior doctors before they have applied for residency in both medical schools in Slovenia, namely, Ljubljana and Maribor. The study was conducted between 12.12.2022 and 8.1.2023. This was a prospective cross-sectional study.

### Questionnaire

The study did not require approval from the Ethical Review Board (ERB) following consultation with the ERB head, as the data collected are nonidentifiable and participants provided informed consent [23]. Informed consent was obtained from each participant on the landing page of the survey. The survey invitation is included in Additional file 1. The information provided to participants included the information about the personal data gathered, the purpose of the study, information about the primary investigator, and information on data handling in accordance with the FAIR (Findable, Accessible, Interoperable, and Reusable) principles. Measures to prevent unauthorized access included the use of a single sign-on solution for logging into the survey environment, 2-factor authentication for accessing the dataset stored on OneDrive, and end-to-end encryption for the transmission of datasets and exports between investigators.

The development of the questionnaire was informed by reviewing manuscripts available online that have previously explored this field [7, 8]. Additionally, we consulted with students who regularly volunteer in the EM department to gather their perspectives and identify important aspects. After drafting the questionnaire, we sought feedback from a survey expert, who provided valuable commentary. Subsequent to incorporating these amendments, we conducted a pilot test with a group of 15 individuals. Their feedback focused on the survey’s flow and offered suggestions on how to rephrase questions to elicit the most informative responses. Following these refinements, the questionnaire was fielded.

The survey was made publicly accessible and did not require a password for entry. Participants were invited via institutional email (students), social media groups (reaching both students and physicians), and a mailing list (physicians). The text of the survey announcement can be found in Additional file 1.

The survey was conducted using 1KA Survey (v 22.10.05, RRID:SCR_019283), an online survey tool developed by the Faculty of Social Sciences, University of Ljubljana [24]. Participation in the survey was voluntary, and no incentives were offered. To encourage engagement, we highlighted that the survey results would be used to formulate recommendations for the ministry on improving EM residency recruitment. The questions in the survey were presented in a fixed order, and adaptive questioning techniques were not employed.

The questionnaire [see Additional file 2] had 18 closed-ended questions regarding the students’ interest in EM residency and different factors that would make EM residency more alluring for medical students. There were also two open-ended questions to determine students’ personal opinions of EM residency and what would absolutely convince the students to choose it. On the first page, there was an introduction and informed consent, followed by a second page with 6 demographic questions (gender, faculty of medicine, level on the path of education, year of birth, postcode of municipality living in, and postcode of municipality where participant want’s to work), on the third page there were 5 questions, of those 4 closed-ended asking about the choice of EM, an open-ended question about the reason for this answer and 3 questions asking the participant to show which specialties are his or her 1st, 2nd and 3rd choice. On the fourth page, we used a 5-point Likert scale from Strongly disagree to Strongly agree on the importance of 11 factors. On the fifth page, there was a question about what would change their mind to choose EM residency. On the sixth 5 closed questions asked about the appropriate base (without overtime and bonuses) net monthly salary for residents and specialists, preferable shift length, worry about stress and complexity of work in EM, and whether their parents work in EM. The seventh question had 8 items that would positively change their mind in choosing EM residency on a 5-point Likert scale from Definitely no to Definitely yes. The ninth page was a thank you note for participation. Altogether, there were 9 screens.

A completeness check was performed through a soft warning (allowing us to proceed without filling out the missing question) for any missing question in a page-by-page notification. There was no review step, but respondents were able to change their answers through the “back button” before submitting their answers.

Unique site visitors were not followed. Rather, the response rate was calculated from the total number of medical students (*n* = 1920) and young doctors (without residents and specialists, *n* = 223) in Slovenia at the time that the survey was carried out.

Cookies were not used to identify the client’s computer. An IP check was used, and the IP address was stored separately and unpaired from the answers. One IP address was allowed to submit one answer per 24 h. No other techniques for log file analysis were used.

Both completed and noncompleted questionnaires were used in the analysis. The sample size is evident in each analysis. Timeframe and atypical timestamps were not used in the analysis. The dataset was not weighed.

### Data analysis

Each question was analyzed separately by first determining the number of respondents who answered each question. To keep as many answers as possible, we kept the respondents who answered each question up to the question we were analyzing. Demographic characteristics and most closely related questions were summarized and reported as frequencies and percentages between genders and levels of respondents’ education.

The basic analysis of the dataset was performed with Python (version 3.10.4) and the help of open-source Python libraries NumPy (version 1.22.3, RRID:SCR_008633) and Pandas (version 1.4.2, RRID:SCR_018214) used for scientific computing. This gave us the frequencies and basic calculations of the mean, median, confidence intervals, skewness and kurtosis. Statistical significance was computed using the Chi-square statistical test with the contingency table function ch2_contingency from the Python statistical functions module (scipy.stats) from the Scipy open source library (version 1.8.1, RRID:SCR_008058). The correction parameter was set to False, where the degrees of freedom were 1. Comparisons by gender were performed using the Mann‒Whitney-Wilcoxon test.

In addition, we used SPSS (v28.0.1.1, RRID:SCR_002865) for part of the analysis where the dimensionality of the question on intention of undergoing EM residency (V6) was reduced with Principal Component Analysis (PCA) with Varimax rotation. Spearman correlation coefficients were computed with the resulting dimensions, gender, age, level of challenge and stress needed by working as an EM physician and likelihood of applying for EM residency. The Cronbach’s α was calculated using the reliability function in SPSS. The result significance was set at *p* < 0.05.

### Purchasing power parities

To make the responses to salary questions comparable with other countries, a conversion from EUR to international dollars (I$) was made by dividing the amount in Slovenian national currency (EUR) by Purchasing Power Parities (PPP), which eliminates differences in price levels between countries and enables purchasing power comparisons [25]. The PPP exchange rate used was sourced from the Organization for Economic Co-operation and Development using data for 2022 (PPP = 0.559), and when converting results from other research, we used PPP according to the year the data were gathered [26].

### Free text analysis

Text analysis was performed by categorizing responses by hand according to their main concept and then segregating them into two distinct groups: the first group encompassed participants who opted for “absolutely yes” and “yes,” while the second group comprised those who selected “absolutely no” and “no.” Subsequently, we conducted a comparison of the percentage of specific categories within each group.

## Results

### Demographics

The overall response rate was 40.1%, but it differed between different demographic groups (Table 1). The number of unique visitors on the first page was 859. The participation rate was 85.8% (589 first-question page clicks/686 first-page clicks). The completion rate, calculated from participants agreeing to participate (*n* = 686) and those who finished the survey (*n* = 436), was 63.6%. Of at least 2143 units in the population (without secondary and ward physicians whose numbers are not tracked by any registry), 676 have started to fill out the survey, and of those, 436 have finished and submitted a complete form (Table 1). Comparing the significance of the subgroups in our sample, we found that there was a statistically significant difference between the first years (compared to the actual proportions in Slovenia). Note that gap year and interns were merged and secondary and ward physicians based on average age. [see Additional file 3– Table 1].

**Table 1:** Sample demographics (NA: not applicable)

	Population size	Started the pool (% from actual group members)	Finished the pool (% from started the pool)
Male	619	191 (31%)	140 (73%)
Female	1524	485 (32%)	296 (61%)
Other gender	0	3 (NA)	0 (NA)
1st year	399	72 (18%)	41 (57%)
2nd year	370	76 (21%)	47 (62%)
3rd year	270	69 (26%)	52 (75%)
4th year	254	86 (34%)	52 (60%)
5th year	271	81 (30%)	50 (62%)
6th year	236	72 (31%)	54 (75%)
Gap year + interns	296	107 (36%)	76 (71%)
Secondary + ward physicians	NA	89 (NA)	64 (72%)
**Total**	**2143**	**679 (31**%**)**	**436 (64**%**)**

### Choice of emergency medicine residency

In response to inquiries regarding their decision to pursue an EM residency, 4% of participants responded with a definitive affirmative, while 11% indicated a probable affirmative. Conversely, 57% expressed a probable negative response, and 28% responded with a definitive negative response (*n* = 515). Due to the low number of respondents who reported that they will select EM (*n* = 17), in further analyses, we merged this category with those that responded they will probably select it, giving us three categories: Definitely no (-2), probably no (-1) and probably or definitely yes [1]. This variable is negatively but moderately associated with age (Rho=-0.337, *p* < 0.001), while no significant differences were found between genders [see Additional file 4– Fig. 1].

Looking at the choice of EM residency over levels of education, the EM as the first choice falls substantially when young doctors first employ after college. A steady decline over levels of education can be seen, with levels in the 1st year of medical school never being achieved again later on, except as a second choice [see Additional file 4– Fig. 2].

**Fig. 3:**
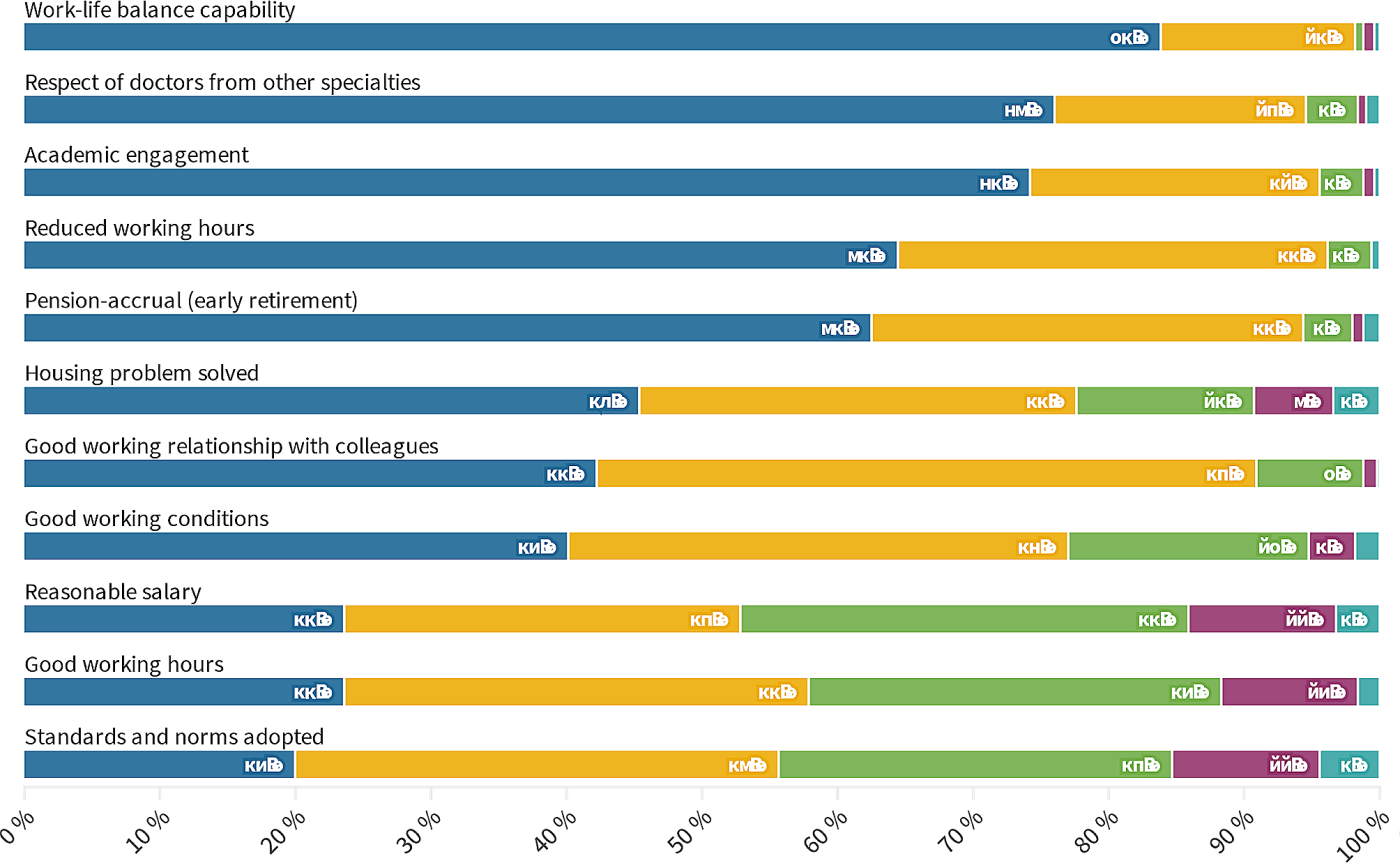
What do the respondents deem important, not filtered by choice of EM residency

Comparing the concerns over job complexity and stress in the EM specialty [see Additional file 4– Fig. 3] showed that those who would choose EM are generally less worried than those who choose other specialties (*p* < 0.001). The likelihood of choosing EM residency is negatively but moderately correlated with the perception of complexity and stress (Rho=-0.337, *p* < 0.001). In addition, we found that the perception of complexity and stress was higher for female than for male students (Rho = 0.211, *p* < 0.001), while there was no significant association with age.

Following exploration into the maximum number of hours per shift, it was discovered that a majority of respondents (58%) favored a maximum of 12 h at a time. In contrast, fewer individuals preferred shifts of 8, 24, and 32 h, with percentages of 26%, 14%, and 1%, respectively (*n* = 445).

**Fig. 4:**
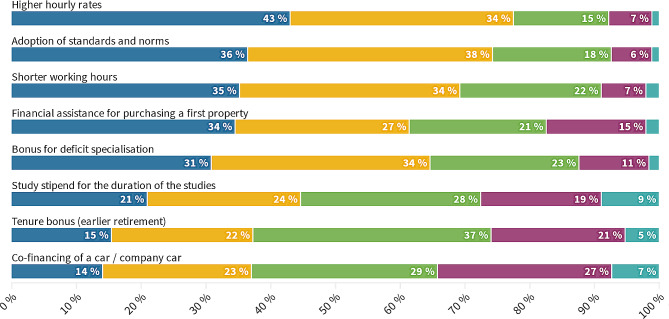
What would change your mind to choose EM residency?

The inquiry regarding the fundamental salary exclusive of benefits for residents and specialists revealed that a majority of respondents concurred on a monthly base salary range of I$ 3623–4529 for residents and I$ 5435–6341 for specialists [see Additional file 4– Fig. 4].

Subsequently, we inquired about individual priorities, revealing that the top three priorities are the attainment of a satisfactory balance between professional and personal life, the demonstration of respect from fellow physicians, and active involvement in academic pursuits. As illustrated in Fig. 3, the factors deemed least significant were the establishment of appropriate working hours, a fair compensation package, and the implementation of work standards and norms (Cronbach’s α = 0.736). In the next section, PCA is used to reduce the dimensionality of these factors.

An inquiry was made as to the factors that would influence the respondents’ decision to select a residency program in EM (Cronbach’s α = 0.833). The three most significant factors were elevated hourly salary, adherence to established standards and norms, and reduced number of working hours. Here, we discerned factors into those that are important (strong agreement > 40% strongly agree) and those that are not. With this, we could form an important specter. There is a significant contrast between individual priorities and those that are considered to be of great importance. As shown in Fig. 4, the study stipend for the duration of studies, pension accrual (early retirement), and co-financing of a car were ranked as the three least significant factors.

### Principal component analysis

To reduce the dimensionality of the evaluation of the importance of eleven different job aspects that are presented in Fig. 3, we ran principal component analysis (PCA) with Varimax rotation with Kaiser normalization that resulted in three components with an eigenvalue over one. Together, they explain 56.52% of the variation, and Table 2 presents their component weights for different items. The first component was named Working conditions, as it has high weights (bolded) for Appropriate working conditions, Appropriate workday, work-life balance capability, Appropriate salary, Accepted standards and norms, and Relation with colleagues. The second component has high values for Academic engagement, Respect from doctors of other specializations, and Housing problems solved; thus, we named it Prestige. The third component has a high weight only for pension-accrual and shortened workdays and was named free time (Table 2).

**Table 2:** PCA components

	Working conditions	Prestige	Free time
Accepted standards and norms	**0.548**	0.337	-0.032
Appropriate salary	**0.732**	0.145	0.136
Appropriate workday	**0.810**	-0.081	0.163
Appropriate working conditions	**0.830**	0.127	-0.021
Relations with colleagues	**0.397**	0.337	0.078
Housing problem solved	0.221	**0.620**	0.267
Pension-accrual	0.052	0.182	**0.884**
Shortened workday	0.179	0.026	**0.882**
Academic engagement	-0.053	**0.742**	-0.048
Respect from doctors of other specialties	0.106	**0.714**	0.093
Work-life balance capability	**0.773**	-0.003	0.155

**Fig. 5:**
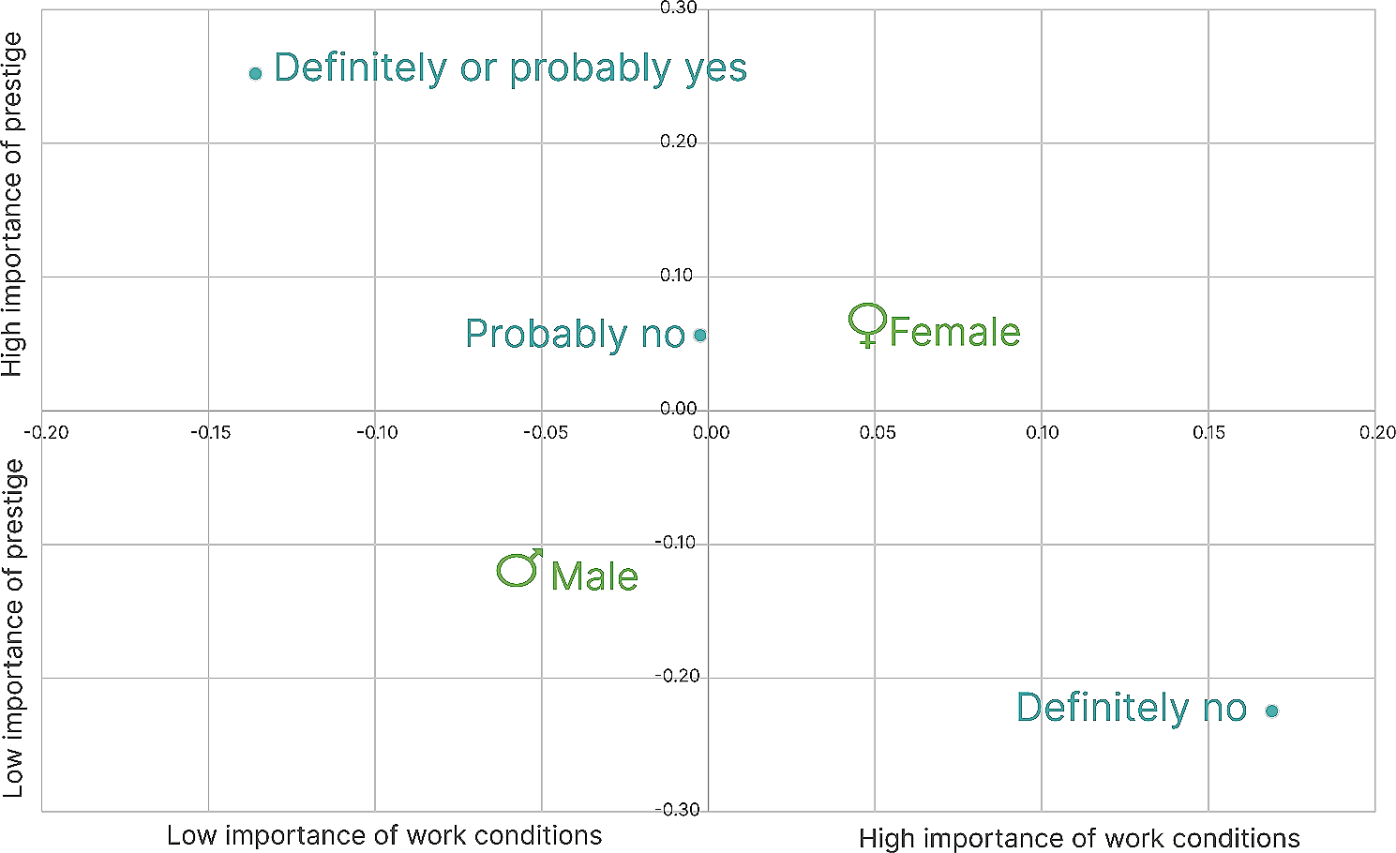
Positioning of centroids for gender and plans to specialize in EM on the working conditions and prestige dimension (the colours represent a single grouping of the variable)

Based on the values of these three dimensions, we calculated centroids as the means for gender (V1), university (V2), year of study (V3), plan to specialize in EM (V6), and desired specialization (V8). For the first dimension, we found significantly higher importance of working conditions among those who definitely do not plan to select EM specialization compared to those who probably or definitely do (F = 5.626, *p* = 0.004), but no significant differences were found for other variables. Groups according to intention to select EM also differed in the second dimension: prestige is significantly highly valued among those who definitely or probably choose EM compared to those who probably or definitely will not (F = 5.580, *p* = 0.004). Moreover, prestige is significantly more important among females than males (F = 5.712, *p* = 0.004) (Fig. 5), while there were no significant differences between means for university, year of study, and desired specialization. Finally, no significant differences between any of the groups were found in the importance of the third dimension, free time.

While there were no significant differences between years of study, a weak but positive correlation was found between age and the importance of free time (Rho = 0.155, *p* = 0.001), and a weak negative correlation was found between age and the importance of prestige (Rho=-0.101, *p* = 0.025). However, no association with work conditions was found.

As mentioned at the beginning of the section, there is a moderate negative correlation between the likelihood of selecting EM residency and age and concern regarding complexity and stress. Moreover, we found a low negative correlation with work conditions (Rho=-0.119, *p* = 0.008) and a weak positive correlation with prestige (Rho = 0.138, *p* = 0.002). In summary, students who are younger, less worried about challenges and stress, and give higher importance to the prestige of their job and lower importance to working conditions are more likely to specialize in EM.

### Open question response analysis

The primary objective of the preliminary investigation was to ascertain the underlying reasoning behind the choice to undertake or abstain from participating in an EM residency program. The descending order of percentage was identical for the first 4 factors in both groups. As shown in Additional file 3– Table 2, both groups identified personal interest, working conditions, and working schedule as the most important factors influencing their decision to choose EM residency. The group that was not planning to do EM residency had family life as the 5th most important factor, followed by mental health. Higher pay, work flexibility, and better education were the least important for both those in favor and those against.

Ultimately, we sought to inquire through open-ended responses what factors would unequivocally prompt participants to select a residency program in EM. The trend exhibits similarity, as the top three factors are shared by both groups. This time, we gave higher importance to those who chose not to undertake EM residency, as that is the group that needs to change their mind to improve the number of applicants. They found that working conditions and system improvement are the top 2 most important factors, while the 3rd most important answer was nothing would convince me. Higher salaries were the 5th most important factor. For those who already favor EM residency working conditions, system improvement and higher salaries were the most important factors [see Additional file 3 - Table 3].

## Discussion

### Current state

Our study found that the intention to choose EM residency matches actual residency applications in Slovenia, as only 4% of the sample is certain to choose EM, compared to 8.2% in Australia [27]. Between 2019 and 2022, EM was the 8th most applied for residency in Slovenia, but when considering the demand for EM residents, it comes in 31st, resulting in only 85% capacity being filled [11]. Similarly, the Australian College for EM is warning that 30% of EM junior doctor positions are set out to be unfilled in 2023 [28]. Between 2022 and 2023, US EM residency match applications dropped 26% [29]. Even worse, 1% of Jordanian medical students chose EM [30]. This suggests a global shortage of EM doctors, highlighting the need to fill these positions.

Despite the previously described gender’s strong influence on residency choice [8], our sample showed no significant gender differences. In other studies, men preferred general surgery, orthopedics, neurosurgery, and EM, while women preferred obstetrics & gynecology, pediatrics, and dermatology [31]. EM interests more men than women, according to a systematic review [8]. With women gaining higher percentages in the EM work environment over time [32], interest in EM has moved from predominantly males to equal parts of both genders. We had not found a study replicating or explaining gender nondifferences, possibly because journals do not publish nonresults. Nevertheless, some gender differences were observed for factors that affect EM choice. Our findings show that females value prestige more than males. However, women perceive more job-related stress than men, which may offset the prestige effect. This opposes prestige, neutralizing gender disparity.

We found that interest in EM decreases over time, peaking in the first year of medical school and reaching its lowest point when young doctors start working in EM. This is further confirmed by a negative correlation with age. Only one study tracked medical students’ interest in specialties over time. A similar result of losing interest over time in medical school has been observed in general surgery residency [33]. The study found that the choice of residency in the final years of medical school strongly correlates with the actual residency choice, which is concerning given our results [34]. A study found that medical students were uncertainir specialty choices at the start but were quite certain by the time of graduation. Nearly three-fourths switched specialties between freshman and senior years [35].

EM doctors are stressed by high patient volumes and rapid throughput [36]. EM is objectively more stressful than other medical specialties in prehospital and hospital care [37]. Our results also show that those who perceive EM as more stressful are less likely to choose EM residency. Extroversion, agreeableness, and conscientiousness reduce stress perception [38]. This suggests that selecting EM residents should include a “right stuff” factor. This finding also underlines the importance of not forcing young doctors to choose a specialty that is not suitable for their personality based solely on economic and care network needs. Our findings show that the general population and EM residency applicants differ in stress and job complexity concerns. Showing that participants have enough introspection to discern if that is an environment for them or not.

### Describing the incoming workforce

The second part of the questionnaire field-tested ideas to attract more applicants, starting with work shift duration. Most preferred a 12-hour workday, with less than half choosing 8 h and even fewer choosing 24 and 32 h. EM residents prefer 9–12-hour shifts, according to a study [39]. Our results are more in favor of 12 h, while Steele et al.’s results are more toward 10 h [39]. Our questionnaire was not granular enough, and our study surveyed a younger population with a lower share of active parents, which has been shown to affect shorter shift choice [40]. Another study found conflicting results, with their sample of EM residents strongly preferring 8 h over 12 h of shift length [41]. Twelve hours of shift length means working fewer days; on average, only 3 days/week, residents have more time to have a better social life, which is a protective factor for well-being [42]. This field needs more research to determine the optimal shift length.

For job seekers, salary is crucial. It is important to know how we compare to other countries and what the next generation expects to keep doctors inside the country. The respondents have suggested base salaries of I$ 43,476 − 65,149 for residents and I$ 65,220 − 76,092 for specialists. From I$ 11,445 in Poland to I$ 293,476 in Luxembourg, specialist gross salaries vary widely across the EU (data from 2015). Ireland, the Netherlands, Denmark, Iceland, and Luxembourg pay their specialists over I$ 150,000 annually. In the UK, GPs earned I$ 105,928, while specialists earned I$ 196,789 [43]. Specialists earned I$ 67,942 in Latvia and I$ 187,600 in Germany in 2020, the latest year with data [43]. In 2019, Slovenian specialists earned I$ 97,587, and general practitioners earned I$ 98,848 [44]. Because of the dispersed data sources, it is difficult to draw conclusions. No one location has the same methodology for tracking the salaries of specialists and residents, as the Organization for Economic Co-operation and Development (OECD) only tracks salaries for specialists, GPs, and hospital nurses [45]. Residents’ and presidency doctors’ salaries are not tracked at all. Additionally, we have asked only about base salary, without over time and bonuses, while OECD tracks average full income, making these values harder to compare.

As the gap between health system needs and doctor supply widens, personal traits and specialty choices are a growing research topic worldwide. This issue affects Slovenia, and local evidence is scarce. In-depth analysis was carried out only for certain specialties, such as radiology [46], general practice [47] and surgery [33], or effects of gender [8], not encompassing a wide spectrum of other specialties in existence. In Australia and New Zealand, a well-developed registry uses longitudinal surveys to collect data on different generations over time [27, 48]. Since research suggests that values differ between generations, the first question on individual priorities was created to identify what important factors influence the specialty decisions of students and young doctors [49]. Our parents’ priorities may not be ours, and vice versa.

We found that there is a specter of importance, ranging from very important to not important. With the most important being work-life balance, which has been identified as important by multiple studies [8, 50–52]. As more people prioritize family over career, current work models must be updated to meet this need [52].

As attitudes of secondary importance, we have identified respect for doctors from other specialties and academic engagement. The Swedish research group has found the research opportunities to be linked with nonsurgical specialties and family medicine [51], while an Australian-New Zealand registry has found that students’ choice is affected by the opportunity for research ranking in 16th place of importance [48]. Respect from other doctors is even more important than work-life balance, according to a UK study [47].

In the tertiary group, we identified reduced working hours and pension accrual. The report from the Australian-New Zealand collaboration has deemed working hours less important, ranking only in 11th place [48]. No literature addresses pension accrual and specialty choice. When analyzing open answers from participants, worry on how to safely conduct work in older age was pointed out multiple times, suggesting that this is a topic in need of more research to strike the desired effect of getting more people excited for the residency in EM while making it sustainable, with a study reporting problems with early retirement in specialty with scarce workforce [53].

Nonpriorities were housing problems solved, relationships with other colleagues and good working conditions, good working hours, reasonable salary-explored above, and standards and norms adopted. This is the complete opposite of an Australian registry survey [48]. This study covers Generation Z, born between 1995 and 2010 [54]. An important characteristic is human-to-human interaction, which is often preferred to take place over a digital medium. This can lead to underdeveloped social and relationship skills and increased risk for isolation, insecurity, and mental health issues, such as anxiety and depression [54]. Work culture/atmosphere was the second most important factor of nonpriorities. The variance between clinical rotations during medical school and internship experience may account for this. Students lack detailed knowledge and job responsibilities beyond their educational scope. Further research is needed in this underresearched area of medical education. Salary was similarly ranked lower. The registry also gave medium importance to working hours, while we found them nonimportant [48]. Researchers have suggested free housing to attract doctors to rural areas [55]. With standards and norms being dead last in importance, this opens an interesting paradigm that needs to be explored, as we did not find any other researcher exploring this as a factor in the choice of specialty. Slovenia has yet to accept standards and norms for work in EM, while it already has them for family medicine, although they have yet to be implemented [56].

### Affecting preresidents’ choice into emergency medicine residency

We hypothesized in the planning phase that personal attitudes may differ from factors that could change residency choice. Contrary to personal attitudes, factors that would change participants’ minds to choose EM residency show almost a complete pivot, a novel and unexplored way of researching career choices in this specialty. This can pave the way for evidence-based policy creation.

Higher working salaries are the most important factor, as discussed above. Here, we will compare their importance. The American Medical Colleges’ graduation questionnaire ranks salary expectation 4th out of 9 [57], and the Australian registry has found it to be only 19th out of 24 factors [48]. EM residency was also positively correlated with salary increase expectations [58].

The adoption of standards and norms (an attentive reader will notice that this annuls the personal factors above), shorter working hours, financial assistance for buying the first property, and bonus for deficit specialization were all given high importance. Standards and norms for EM already exist under the World Health Organization [59] but have yet to reach most countries, making this an important finding for all countries trying to enroll more residents in EM. Shorter working hours have been covered above. Working hours have been important to UK doctors since 1999 [60]. With a high ranking in our survey, financial assistance for first-time homebuyers warrants further study. Bonuses are a common way to make deficient jobs more appealing. Family medicine is a deficitiary in Slovenia, giving residents a 20% bonus [61]. This failed, with only 27/100 spots applied for in the 2023 first call [11].

Study stipends, pension accrual, and company car/car co-financing are the least important parts of the specter. Medical school stipends and specialty choice research are scarce. We found that cardiothoracic surgery retained one-third of all student recipients to choose it and pursue a career in it [62]. A larger study found that stipends for medical students had a 2.34 odds ratio for choosing rural medicine, encouraging more students to pursue that career [63]. Pension accrual was discussed above.

We set out to identify important factors by binding them together and performing PCA. The German study’s components were similar to ours: financial and bureaucratic barriers, leisure time and work-life balance, and rural medical practitioners’ isolation [50]. Our components one and three mostly match component two from the study, suggesting that medical students’ priorities may be similar across Europe. A US medical school study on internal medicine specialty choice found prestige and lifestyle to be important factors [64]. Our study’s prestige component is linked to women, contrary to the Baxter et al. 1996 study, suggesting further research [65].

The components in PCA have then been used to compute Spearman correlation coefficients, and we have found that the importance of prestige positively but weakly correlates with the choice of EM, while it negatively but weakly correlates with the importance of work conditions. This is contrary to the Singaporean medical students, who had prestige (measured directly, unlike our study where we measured it through PCA) positioned as a negative factor of influence [66]. Age correlated negatively, which also translates to actual applications for EM residency in Slovenia [11]. The third significant component was worry about the level of challenge and stress, which was negatively correlated with the choice of EM and is covered in depth below.

Finally, the participants answered two unstructured questions about why they would (not) choose EM residency and what would definitely change their minds to choose it. Personal interest was the main reason for choosing and not choosing EM residency, consistent with other studies [8, 60]; this is the group we cannot persuade to change their mind. In line with other research, working conditions, schedule, and stress were secondary factors [48]. EM candidates should also be stress resistant [38], and choosing a surgical specialty is linked to this trait as well [67].

Working conditions and system improvements dominated the second open question. The physical environment is important. Our patients and staff often work in crowded, unsuitable environments for modern EM. They notice the contrast to other islands of tranquility within the acute hospital and wider health economy. The ED must be conducive to high-quality health care [12]. We investigated and compared our system to other EU countries. Knowing that 31% of ED visits are nonemergency, most EDs strain from them [68, 69], causing overcrowding and stress [70]. Initially, we compared the number of EU general practitioners. In 2017, Slovenia had 76.75 GPs per 100,000 people, eighth in the EU, followed by Poland at 41.89 and behind Portugal at 262.87, Denmark had 161.87 [71]. Understanding national age distribution is crucial. Slovenia ranks 11th in the EU by its elderly population. Approximately 21% of the country’s inhabitants fall within this age category. Portugal has a higher elderly population than Slovenia, with 23% over 65. Denmark has 20% of elderly people [72]. Due to their similar age structures, we compared the Portugal and Slovenian EM systems. The evidence on the number of ED visits and primary care network accessibility is conflicting and still requires further investigation [73]. By reforming Portuguese primary health care (PHC), they have shown that improved accessibility and continuity of care can enable patients to receive adequate healthcare from PHC facilities, reducing their reliance on EDs except in emergencies [73]. Denmark has similar attributes as Portugal, with it additionally having an EM specialty. Denmark discourages patients from going to the ED without first calling their GPs, who can assess them over the phone, refer them to a GP visit, send a GP to their home, or send them to the ED [74]. This results in high inpatient admissions and short lengths of stay, better utilizing the ED and ensuring that it is not overcrowded, even though this system still reports intensive care unit overcrowding at least monthly [74].

Slovenia’s palliative care plan, which is only now being implemented, is another issue that drives ED use [75]. These and PHC improvements are crucial to alleviating system constraints that deter EM residency applicants.

The third most common theme was nothing would convince me, which is not surprising given the results of the first open question and other research [8, 60]. Management and system were the fourth reason, with salaries fifth.

Only 4.5% of respondents worried about working as an EM specialist as they got older. In contrast, 76% of French students found it difficult to practice for an entire career [76].

Open-ended questions show that work schedule and conditions matter more than salary, which is the opposite of structured questions. We attribute the nondeniable difference to the fact that the structured part of the questionnaire already collects the most important factors and open questions just provide context and fill in the gap if the structured answers were unavailable. More participants answered structured questions than unstructured ones.

Our findings on career choice in emergency medicine residency show that in accordance with social cognitive career theory [1], personal factors such as individual interests and stress tolerance levels have emerged as significant determinants in choosing an EM residency. Environmental factors [1], including labor market conditions such as salary expectations, availability of financial incentives, and work-life balance, also play a crucial role. These external conditions, reflecting the labor supply and demand dynamics, can either enhance or limit the attractiveness of EM as a career path. Furthermore, the comparative analysis of health systems across countries such as Slovenia, Portugal, and Denmark provides an understanding of how broader environmental factors, such as national health policies and primary care accessibility, can influence individual career choices in EM. For future studies, a longitudinal approach focused on the results of systemic reforms targeting working conditions, salaries, and other financial incentives should be used.

### Limitations

The lack of a standardized questionnaire restricts the comparability of our study. International cooperation should create a standard for better comparisons. Our study only examined one point in time; thus, we cannot determine how attitudes and preferences change over time. We could achieve better temporal resolution by creating a panel doing this kind of inquiry yearly. Another intriguing possibility is looking at how the year’s subject list affects attitudes toward specialties. Our questionnaire did not include personality measures, but previous studies did. Our shift length question was too broad, encompassing individual brackets that were too long. This allows us to show only rough outlines. A more granular questionnaire would help determine the best shift length. The PPP used was from 2022, even though the study spanned between 2022 and 2023, which was due to the timing of the research, as it was carried out between December 2022 and January 2023. At the time of writing, PPP for 2023 was not yet available. The sampling technique was random inside the subgroups that were chosen. This could lead to the sample not representing the actual state, as not all of the subgroup members were surveyed, and the results could be biased toward the persons who were more likely to take part in our survey.

## Conclusions

Our study suggests that improving compensation, setting norms and standards, improving work-life balance, aiding in property acquisition, encouraging residency program participation, fostering mutual respect among colleagues, limiting shift duration, and enabling pension accrual attract more candidates for EM residency. EM requires a specific “right stuff” attitude, which not everybody has. Physicians should not be forced to apply for EM. Personal attitudes differ from factors that influence an individual’s choice of specialty, a novel phenomenon. This suggests that future studies should include both questions. System reform is crucial. Instead of addressing issues in specific fields, departments, or hospitals, a comprehensive strategy is needed.

### Electronic supplementary material

Below is the link to the electronic supplementary material.

Supplementary Material 1

Supplementary Material 2

Supplementary Material 3

Supplementary Material 4

## Data Availability

The datasets generated during and/or analyzed during the current study are available in the Slovenian Social Science Data Archives repository repository, Petravić, L., Burger, E., Tiefengraber, E. and Strnad, M. (2023). Attitudes toward emergency medicine residency in Slovenia, 2023 [Data file]. Ljubljana: Univerza v Ljubljani = University of Ljubljana, Slovenian Social Science Data Archives (ADP). ADP - IDNo: MSUM23. 10.17898/ADP_MSUM23_V1.

## Abbreviations

ED: Emergency Department
EM: Emergency Medicine
ERB: Ethical Review Board
EUR: Euro currency
FAIR: Findable, Accessible, Interoperable, and Reusable
GP: General Practitioner
GDP: Gross domestic product
HIIS: Health Insurance Institute of Slovenia
I$: International Dollar
IP: Internet Protocol address
NA: Not Applicable
OECD: Organisation for Economic Co-operation and Development
PHC: Primary Health Care
PCA: Principal Component Analysis
PPP: Purchasing Power Parity
RRID: Research Resource Identifier
SPSS: Statistical Package for the Social Sciences
UK: United Kingdom
US$: United States Dollar
US: United States of America
