# Supplementary material for: Emergency physician personnel crisis: a survey on attitudes of new generations in Slovenia
Source: BMC Emerg Med. 2024 Feb 14;24:25. doi: 10.1186/s12873-024-00940-z (PMC10865631; doi:10.1186/s12873-024-00940-z)
Supplement: Supplementary file 1 — Supplementary Material 1 [file 12873_2024_940_MOESM1_ESM.docx]

# Additional fille 1 – Survey invitation

[ENGLISH - translation]

Dear colleagues!

If you have already completed the survey in full (only fully completed surveys will be analysed), please skip this message and thank you for your participation.

If you have not yet completed the survey, please do not hesitate to participate.

As we have recently seen a decline in applications for specialisation in emergency medicine (and at the same time the NMP reform is taking place), we are interested in what would attract young doctors and what would influence their decision to choose specialisation in emergency medicine. Therefore, I would like to ask you to complete the survey (link below) in as large a number as possible to ensure that the sample is representative.

https://1ka.arnes.si/urgentna_2

Data collection and processing will be anonymous. Your participation will enable or help to create the conditions that will make it easier for colleagues to decide to specialise in emergency medicine.

Thank you for your understanding and cooperation.

All the best in 2023.

Matej Strnad, MD, PhD

Assoc. Professor

Community Healthcare Center dr. Adolf Drolc Maribor, Emergency Medical Service Maribor, Prehospital Unit

University Medical Center Maribor, Emergency Department

University of Maribor, Medical Faculty, Head of the Department of Emergency Medicine
