# Supplementary material for: Emergency physician personnel crisis: a survey on attitudes of new generations in Slovenia
Source: BMC Emerg Med. 2024 Feb 14;24:25. doi: 10.1186/s12873-024-00940-z (PMC10865631; doi:10.1186/s12873-024-00940-z)
Supplement: Supplementary file 2 — Supplementary Material 2 [file 12873_2024_940_MOESM2_ESM.docx]

# Additional file 2 – questionnaire

[ENGLISH – translation]

Consent to the collection of personal data in the survey The survey in front of you collects personal data and information that you provide in the survey:

- Other identifiers - year of birth, location of study, year of study, municipality of residence, municipality of desired specialisation.

As we will be collecting the above personal data together with your answers, we ask you to agree to the collection of your personal data before completing the form. The provision of the survey and personal data is voluntary and a condition for participation in the survey. If you do not provide the information, you cannot continue to complete the survey. Details on the collection, storage and processing of your data in this survey can be found here. The privacy policy and terms and conditions are available here.

Please indicate whether you agree to the collection of your personal data:

No, I do not agree to the collection of my personal data

Yes, I agree to the collection of my personal data

Hello,

In front of you is the questionnaire and the opportunity to help shape the policy of the specialty of emergency medicine. We are aware that there are too few emergency physicians in Slovenia and that few people choose this career path.

We would like to change this.

By filling in the survey you will help us understand what your priorities are, what should be changed to achieve higher enrolment in the specialty of emergency medicine.

The survey is anonymous and the data will be used to shape the policy for recruiting new emergency medicine specialists. The survey data will be made publicly available for researchers to analyse.

Participation is voluntary and anonymous. By continuing you agree to participate.

For any questions, please contact me at: matej.strnad@um.si

Thank you in advance for your participation,

Prof. Matej Strnad

Chair of Emergency Medicine MFUM, NMP MB, UC MB.

Q1 - Gender

Male

Female

Other

Q2 - Faculty of Medicine

In Ljubljana

In Maribor

Q3 - Currently attending

1st year

2nd year

3rd year

4th year

5th year

6th year

Graduates

Traineeships

Secondary

Room doctor_ ce

Work as a specialist

Work as a specialist

Q4- Year of birth ____________________

Q5.1 - Postcode of the municipality of your permanent residence

Q5.2 - Postcode of the municipality in which you would like to specialise

Q6 - Do you intend to specialise in emergency medicine?

Definitely yes

Probably yes

Probably not

Absolutely not

Q7 - Why?

__________________

V8.1 - 1st choice spec

Abdominal surgery

Allergy and clinical immunology (adults)

Anaesthesiology, critical care and perioperative intensive care medicine

Dermatovenerology

Family medicine

Physical and rehabilitation medicine

Gastroenterology

Gynaecology and Obstetrics

Haematology

Infectology

Intensive care medicine

Internal Medicine

Internal Medicine Oncology

Public Health

Cardiology and Vascular Medicine

Cardiovascular Surgery

Clinical Pharmacology

Clinical Physiology

Clinical Genetics

Clinical Microbiology

Maxillo-facial surgery

Nephrology

Neonatology

Neurosurgery

Neurology

Nuclear medicine

Ophthalmology

Oncology with radiotherapy

Orthopaedic surgery

Otorhinolaryngology

Child and Adolescent Psychiatry

Paediatric surgery

Child neurology

Pathology

Paediatrics

Plastic, reconstructive and aesthetic surgery

Pneumology

Psychiatry

Rheumatology

Forensic Medicine

General surgery

Thoracic surgery

Transfusion Medicine

Traumatology

Emergency Medicine

Urology

Vascular Surgery

Radiology

V8.2 - 2nd choice spec

Abdominal surgery

Allergy and Clinical Immunology (Adult)

Anaesthesiology, critical care and perioperative intensive care medicine

Dermatovenerology

Family medicine

Physical and rehabilitation medicine

Gastroenterology

Gynaecology and Obstetrics

Haematology

Infectology

Intensive care medicine

Internal Medicine

Internal Medicine Oncology

Public Health

Cardiology and Vascular Medicine

Cardiovascular Surgery

Clinical Pharmacology

Clinical Physiology

Clinical Genetics

Clinical Microbiology

Maxillo-facial surgery

Nephrology

Neonatology

Neurosurgery

Neurology

Nuclear medicine

Ophthalmology

Oncology with radiotherapy

Orthopaedic surgery

Otorhinolaryngology

Child and Adolescent Psychiatry

Paediatric surgery

Child neurology

Pathology

Paediatrics

Plastic, reconstructive and aesthetic surgery

Pneumology

Psychiatry

Rheumatology

Forensic Medicine

General surgery

Thoracic surgery

Transfusion Medicine

Traumatology

Emergency Medicine

Urology

Vascular Surgery

Radiology

V8.3 - 3rd choice spec

Abdominal surgery

Allergy and clinical immunology (adults)

Anaesthesiology, critical care and perioperative intensive care medicine

Dermatovenerology

Family medicine

Physical and rehabilitation medicine

Gastroenterology

Gynaecology and Obstetrics

Haematology

Infectology

Intensive care medicine

Internal Medicine

Internal Medicine Oncology

Public Health

Cardiology and Vascular Medicine

Cardiovascular Surgery

Clinical Pharmacology

Clinical Physiology

Clinical Genetics

Clinical Microbiology

Maxillo-facial surgery

Nephrology

Neonatology

Neurosurgery

Neurology

Nuclear medicine

Ophthalmology

Oncology with radiotherapy

Orthopaedic surgery

Otorhinolaryngology

Child and Adolescent Psychiatry

Paediatric surgery

Child neurology

Pathology

Paediatrics

Plastic, reconstructive and aesthetic surgery

Pneumology

Psychiatry

Rheumatology

Forensic Medicine

General surgery

Thoracic surgery

Transfusion Medicine

Traumatology

Emergency Medicine

Urology

Vascular Surgery

Radiology

Q9 - Please rate how important the following items are to you

Strongly Disagree Disagree Neither Agree nor disagree Agree Strongly agree

Accepted standards and norms

Appropriate salary

Adequate working hours

Adequate working conditions

Relations with colleagues

Housing problem solved

Pension acrual (early retirement)

Shortened working hours

Academic pursuits

Respect for doctors of other specialties

Ability to balance work and family life

Q10 - What would absolutely convince you to specialise in emergency medicine?

Think as broadly as possible.

___________________________________________

Q11.1 - What do you consider to be an appropriate monthly salary for an emergency medicine specialist?

The current starting salary of a specialist is 1215,18 €/month net (excluding bonuses and overtime).

From 1000 to 1500 EUR

From 1500 to 2000 EUR

From 2000 to 2500 EUR

From 2500 to 3000 EUR

From EUR 3000 to EUR 3500

From EUR 3500 to EUR 4000

From 4000 to 4500 EUR

From EUR 4500 to EUR 5000

From EUR 5000 to EUR 5500

From EUR 5500 to EUR 6000

From EUR 6000 to EUR 6500

From EUR 6500 to EUR 7000

From EUR 7000 to EUR 7500

From EUR 7500 to EUR 8000

From EUR 8000 to EUR 8500

From EUR 8500 to EUR 9000

From EUR 9000 to EUR 9500

From EUR 9500 to EUR 10 000

Q11.2 - What do you consider to be an appropriate monthly salary for an emergency medicine specialist?

The current salary of a specialist is EUR 1717.92/month net (excluding bonuses and overtime).

From 1000 to 1500 EUR

From 1500 to 2000 EUR

From 2000 to 2500 EUR

From 2500 to 3000 EUR

From EUR 3000 to EUR 3500

From EUR 3500 to EUR 4000

From 4000 to 4500 EUR

From EUR 4500 to EUR 5000

From EUR 5000 to EUR 5500

From EUR 5500 to EUR 6000

From EUR 6000 to EUR 6500

From EUR 6500 to EUR 7000

From EUR 7000 to EUR 7500

From EUR 7500 to EUR 8000

From EUR 8000 to EUR 8500

From EUR 8500 to EUR 9000

From EUR 9000 to EUR 9500

From EUR 9500 to EUR 10 000

Q12 - How many hours would you work in total in one shift?

up to 8 hours

up to 12 hours

up to 24 hours

up to 32 hours

Q13 - To what extent are you concerned about the high level of complexity and stress that the job of an emergency physician requires?

I am not at all concerned

Quite worried

Very worried

Q14 - Are your parents or other close relatives active in the field of emergency medicine?

Yes

No

Q15 - To what extent would individual actions influence your decision about emergency medicine?

Definitely No No Maybe Yes Definitely Yes

Benificated working hours (previously retired)

Shorter working hours

Higher hourly rates

Deficit specialty allowance

Financial assistance for the purchase of a first property

Adoption of standards and norms

Co-financing of a car / company car

Staff scholarship for the duration of studies
