# Supplementary material for: Emergency physician personnel crisis: a survey on attitudes of new generations in Slovenia
Source: BMC Emerg Med. 2024 Feb 14;24:25. doi: 10.1186/s12873-024-00940-z (PMC10865631; doi:10.1186/s12873-024-00940-z)
Supplement: Supplementary file 3 — Supplementary Material 3 [file 12873_2024_940_MOESM3_ESM.docx]

# Additional file 3 – Additional tables

Additional table 1 Year of birth by group

|  | 1st year | 2nd year | 3rd year | 4th year | 5th year | 6th year | Gap year | Internship | Secundarium | Ward doctor | Resident |
| --- | --- | --- | --- | --- | --- | --- | --- | --- | --- | --- | --- |
| Mean year of birth | 2003 | 2002 | 2000 | 2000 | 1998 | 1998 | 1996 | 1996 | 1995 | 1995 | 1993 |

Additional table 2 Open-question analysis on choice of EM residency, hand categorized.

|  | Absolutely yes and probably yes column percent (n=67) | Absolutely no and probably no column percent (n=381) |
| --- | --- | --- |
| Personal interest | 52.2 % | 21.8 % |
| Working conditions | 37.3 % | 18.4 % |
| Bad schedule | 3.0 % | 15.5 % |
| Stress | 3.0 % | 15.0 % |
| Mental health | 1.5 % | 5.5 % |
| Worrying about work when you get older | 1.5 % | 4.5 % |
| Prejudice | 1.5 % | 4.2 % |
| Family life | 0.0 % | 7.3 % |
| Burnout | 0.0 % | 3.7 % |
| Higher salary | 0.0 % | 2.9 % |
| Work flexibility | 0.0 % | 0.8 % |
| Better education | 0.0 % | 0.5 % |

Additional table 3 Open-question analysis on what would certainly make you choose EM, hand categorized.

|  | Absolutely yes and probably yes column percent (n=67) | Absolutely no and probably no column percent (n=381) |
| --- | --- | --- |
| Working conditions | 41.4 % | 33.0 % |
| Improving the system | 27.6 % | 27.0 % |
| Nothing convinces me | 5.2 % | 15.8 % |
| Better work schedule | 6.9 % | 12.7 % |
| Higher salaries | 12.1 % | 6.1 % |
| Good relationships and mentoring | 1.7 % | 3.9 % |
| Regular training | 0.0 % | 0.9 % |
| More staff | 5.2 % | 0.6 % |
