# Supplementary material for: Emergency physician personnel crisis: a survey on attitudes of new generations in Slovenia
Source: BMC Emerg Med. 2024 Feb 14;24:25. doi: 10.1186/s12873-024-00940-z (PMC10865631; doi:10.1186/s12873-024-00940-z)
Supplement: Supplementary file 4 — Supplementary Material 4 [file 12873_2024_940_MOESM4_ESM.docx]

# Additional file 4 – Additional figures


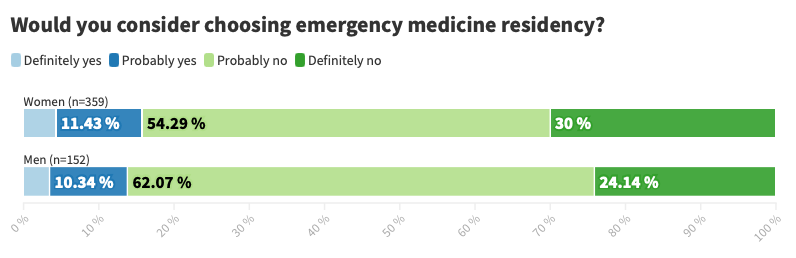


**Additional figure 1 Comparing first choice and cumullative first three choices for residency by gender.**


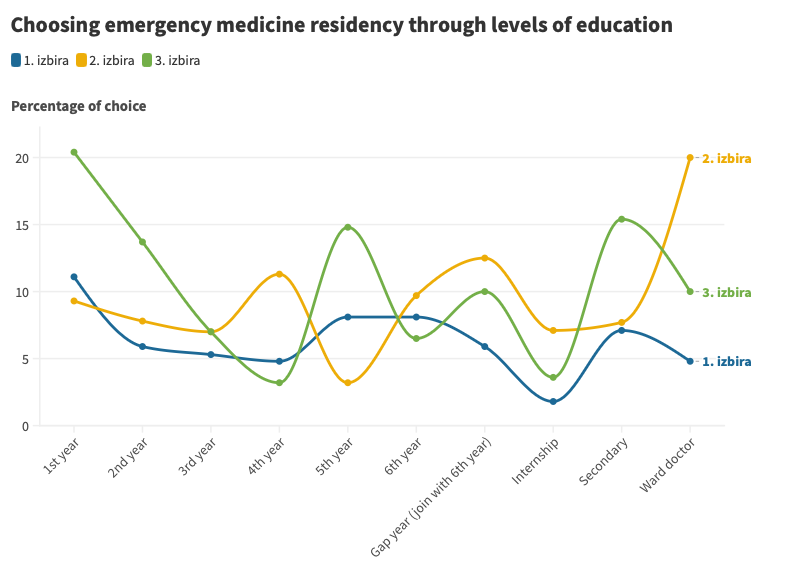


**Additional figure 2 1st, 2nd and 3rd choice of EM residency over levels of education.**


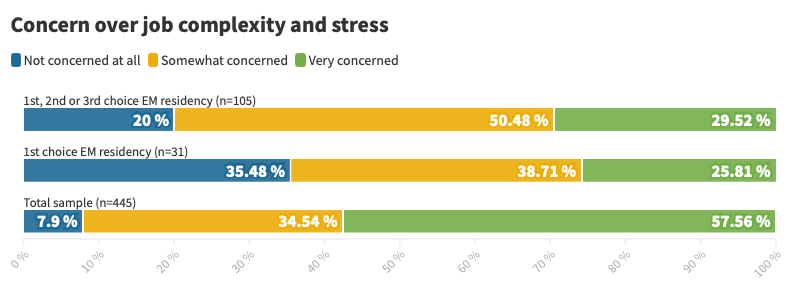


**Additional figure 3 Concerns over EM job complexity and stress.**


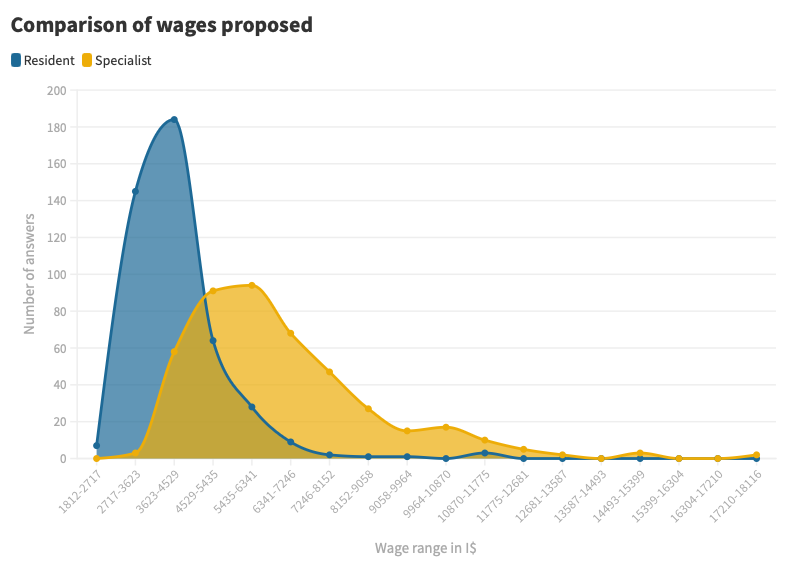


**Additional figure 4 Distribution of votes regarding base sallary of residents (in blue) and specialists (in yellow).**
